# Quercetin and l-Arginine Ameliorated the Deleterious Effects of Copper Oxide Nanoparticles on the Liver of Mice Through Anti-inflammatory and Anti-apoptotic Pathways

**DOI:** 10.1007/s12011-023-03884-w

**Published:** 2023-09-30

**Authors:** Amina M. Haroun, Wael M. El-Sayed, Rasha E. Hassan

**Affiliations:** 1 Department of Zoology, Faculty of Science, Ain Shams University, Abbassia, Cairo, 11566 Egypt; 2 Department of Biochemistry, Faculty of Science, Ain Shams University, Abbassia, Cairo, 11566 Egypt

**Keywords:** Antioxidant, Arginase, Caspase-3, Hepatotoxicity, Oxidative stress

## Abstract

The widespread use and applications of copper oxide nanoparticles (CuO NPs) in daily life make human exposure to these particles inevitable. This study was carried out to investigate the deteriorations in hepatic and serum biochemical parameters induced by CuO NPs in adult male mice and the potential ameliorative effect of l-arginine and quercetin, either alone or in combination. Seventy adult male mice were equally allocated into seven groups: untreated group, l-arginine, quercetin, CuO NPs, arginine + CuO NPs, quercetin + CuO NPs, and quercetin + arginine + CuO NPs. Treating mice with CuO NPs resulted in bioaccumulation of copper in the liver and consequent liver injury as typified by elevation of serum ALT activity, reduction in the synthetic ability of the liver indicated by a decrease in the hepatic arginase activity, and serum total protein content. This copper accumulation increased oxidative stress, lipid peroxidation, inflammation, and apoptosis as manifested by elevation in malondialdehyde, nitric oxide, tumor necrosis factor-α, the expression level of caspase-3 and bax quantified by qPCR, and the activity of caspase-3, in addition to the reduction of superoxide dismutase activity. It also resulted in severe DNA fragmentation as assessed by Comet assay and significant pathological changes in the liver architecture. The study proved the efficiency of quercetin and l-arginine in mitigating CuO NPs-induced sub-chronic liver toxicity due to their antioxidant, anti-inflammatory, and anti-apoptotic properties; ability to inhibit DNA damage; and the potential as good metal chelators. The results of histopathological analysis confirmed the biochemical and molecular studies.

## Introduction

Copper oxide nanoparticles (CuO NPs) are among the most important engineered nanoparticles due to their excellent optical, electrical, catalytic, and antimicrobial properties. CuO NPs are widely used in electronics, ceramics, films, polymers, inks, lubricant oils, and coatings [1]. Additionally, they have shown great promise in the development of antibacterial products, as a supplement in livestock and poultry feed, and as a component of intrauterine contraceptive devices [2]. The widespread use of CuO NPs increases the likelihood of the release of CuO NPs into the environment and the chance of human exposure to these particles [3, 4]. Even though CuO NPs have proven their use in biomedical applications, the major disadvantage of their use in the medical field is due to their potentially toxic effects [5]. Various in vitro* a*nd in vivo investigations clarified that exposure to CuO NPs produces oxidative stress, genotoxicity, immunotoxicity, neurotoxicity, hepatotoxicity, nephrotoxicity, and severe splenic injury, as well as DNA damage and inflammation [6, 7]. The concomitant use of nutraceuticals or botanical metabolites with these nanoparticles could reduce their toxicity and provide a safer platform. In the current study, we investigated the potential protective effects of quercetin and l-arginine.

Quercetin, a polyphenolic flavonoid, is widely found in kale, onions, berries, apples, red grapes, broccoli, cherries, and tea [8]. It is a versatile molecule with a wide range of pharmacological activities, including antioxidant, neuroprotective, antiviral, anticancer, cardiovascular, antibacterial, hepatoprotective, and anti-obesity [9]. Quercetin is also known as an anti-inflammatory/anti-allergy natural remedy, where it stabilizes the cell membrane of mast cells and prevents the release of histamine and other inflammatory mediators in the body [10]. The antioxidant properties of quercetin play an important role in the prevention and treatment of a variety of disorders [8].

l-Arginine (l-Arg) is a semi-essential amino acid involved in multiple areas of human physiology and metabolism. l-Arginine is the source of nitric oxide (NO) in biological tissues produced from its terminal guanidinium group by nitric oxide synthase (NOS). Therefore, arginine possesses a potent anti-stress activity [11], regulates cellular redox status, and plays an effective role against oxidative stress [12]. Several studies have proved that l-arginine has a therapeutic effect on numerous acute and chronic diseases including sickle cell chest crisis, pulmonary artery hypertension, coronary heart disease, and pre-myocardial infarction [13].

Therefore, the current study aimed to investigate the potential hepatoprotective effect of quercetin and/or l-arginine against liver damage induced by copper oxide nanoparticles in adult male mice and explore the associated molecular mechanisms.

## Materials and Methods

### Animals

Seventy adult male mice weighing 20–30 g, obtained from the Faculty of Veterinary Medicine (Giza, Egypt), were housed in steel mesh cages (10/cage) for 1 week for acclimatization under controlled conditions at 23 °C ± 1 °C with a natural 12-h light/dark cycle and 50% ± 5% relative humidity. All mice were fed on standard rodent pellets obtained from the Agricultural Industrial Integration Company, (Giza, Egypt) and had a free access to water. The experimental animals were humanely treated following the Laboratory Animal Care and Use Guide from the National Institutes of Health (NIH Publications No. 8523, revised 2011). The research protocol was approved by the Ethics Committee on Animal Use (Code ASU-SCI/ZOOL/2023/5/1).

### Chemicals

Quercetin (Qu) and l-arginine (l-Arg) were purchased from Sigma (St. Louis, MO, USA) and LOBA Chemie (India), respectively. Copper oxide nanoparticles (CuO: size 20–30 nm, purity 99.5%, spherical in shape, black powder, specific surface 43.2 m^2^/g, phase monoclinic, batch # 123332390) were obtained from ROTH (Germany).

### Experimental Design

Experimental animals were equally divided into seven groups (*n* = 10). Group (1): Control group received distilled water by oral gavage. Group (2): Animals received 100 mg/kg of CuO NPs suspended in distilled water by oral gavage, day after day for 8 weeks [3]. Group (3): Mice received 50 mg/kg of (l-Arg) dissolved in distilled water by oral gavage, day after day for 8 weeks [14]. Group (4): Mice received 50 mg/kg of (Qu) dissolved in warm distilled water by oral gavage, day after day for 8 weeks [15]. Group (5): Mice received the same previous doses of l-Arg and CuO NPs concomitantly. Group (6): Mice received the same previous doses of Qu and CuO NPs concomitantly. Group (7): Mice received l-Arg, Qu, and CuO NPs concomitantly.

### Blood and Tissue Sampling

At the end of the experiment (8 weeks), the animals were weighed and then anesthetized using isoflurane after fasting for 12 h. Blood samples were withdrawn into capillary tubes from the retro-orbital venous plexus. Sera were immediately separated by centrifugation at 5000 rpm for five min, then aliquoted and stored at – 80 °C until analysis. Immediately after sacrifice, liver tissue was perfused, dissected out, and divided into three parts; the first part (~ 0.1 g) was placed in a sterilized microfuge tube and kept at – 80 °C for qRT-PCR, the second part was kept in 10% formalin for histopathological examination, and the third part was rinsed in isotonic saline solution, blotted dry with filter paper, weighed, and homogenized for biochemical assays.

### Preparation of Liver Homogenate

Liver homogenate (10%) was prepared by homogenizing 1:10 w/v of liver tissue in phosphate buffer saline (PBS) pH 7.2. The homogenate was centrifuged at 3000 rpm for 15 min at 4 °C, and then, the supernatant was removed, aliquoted, and stored at – 80 °C, till biochemical measurements.

### Determination of Copper Accumulation in Liver Homogenate

Copper accumulation in the liver was determined by atomic absorption according to the method described elsewhere [16] using Flame Atomic Absorption (GBC Scientific Equipment, Pty Ltd., Australia). The optimum conditions for copper were wavelength: 324.8 nm, acetylene flow rate: 0.5 L min^−1^, air flow rate: 4.0 min^−1^, and slit width: 0.7 nm.

### Biochemical Analyses

Liver function tests were carried out by kinetic measuring of serum alanine aminotransferase (ALT) activity [17] using a kit from BM Egypt (Cairo, Egypt) and a Beckman Coulter analyzer. Total protein (TP) content was determined in serum using a colorimetric assay kit (SPINREACT, Spain) [18]. Arginase activity was determined in liver tissue as described before [19] using a colorimetric assay kit (BioVision, California, USA). Superoxide dismutase (SOD) activity, malondialdehyde (MDA), and NO contents were determined in liver homogenate as described elsewhere [20–22], respectively. The level of inflammatory marker tumor necrosis factor-α (TNF-α) in serum was determined using an ELISA assay kit (CUSABIO, USA) [23]. Caspase-3 activity was detected by a colorimetric assay kit (MBL, Woburn, USA) [24].

### Evaluation of DNA Fragmentation

Comet assay was used to evaluate the CuO NPS-induced DNA damage according to Singh et al. [25]. Briefly, 500 mg of chopped liver sample was gently homogenized in ice cold phosphate buffer saline (PBS) with a homogenizer (Ikemoto Scientific Technology Company Ltd., Japan). The homogenate was centrifuged for 10 min at 4 °C and 15,000 rpm and filtered. A volume of 100 μL of cell suspension was mixed with 600 μL of low melting agarose (0.8% in PBS). Then, 100 μL of the mixture was spread on pre-coated slides. The coated slides were immersed in lysis buffer (0.045 M TBE, pH 8.4, containing 2.5% SDS) for 15 min to eliminate any proteins. The slides were placed in electrophoresis chamber containing the same TBE buffer, but devoid of SDS. The electrophoresis conditions were 2 V/cm for 2 min and 100 mA. Staining with ethidium bromide (20 μg/mL) was performed at 4 °C. The observation was performed while the samples were still humid. The DNA fragment migration patterns were evaluated with fluorescence microscope (with excitation at 510 nm). The comet’s tail lengths were measured from the middle of the nucleus to the end of the tail with 40 × for the count and measure the size of the comet. Komet 5 image analysis software developed by Kinetic Imagining, Ltd. (Liverpool, UK) was used for the image analysis linked to a CCD camera to assess the quantitative and qualitative extent of DNA damage, and the tail moment was calculated by the program from the following equation:

$$\left(\mathrm{DS}3*\left(\left(\left(\mathrm{DS}1-\mathrm{DS}2\right.\right.\right)/2\right)+\left(\left.\left.\mathrm{DS}2/2\right)\right)\right)/100$$

where DS1 was the total comet length, DS2 was the comet head length, and DS3 was the percent DNA in tail value.

### Molecular Analysis

*Caspase-3* and *bax* gene expression levels were assessed in liver tissue by quantitative real-time polymerase chain reaction (qRT-PCR). Total RNA extraction kit, reagents for cDNA synthesis, and SYBER Green Master Mix were obtained from Thermo Scientific (USA). Total RNA was isolated from hepatic tissues by triazole reagent according to the instructions of the manufacturer and subsequent cDNA was synthesized using power first-strand cDNA synthesis kits according to the manufacturer protocol. The primer sequence for the studied genes is illustrated in Table 1.

**Table 1:** Primer sequences for *caspase-3*, *bax*, and *GAPDH*

Genes	Primers	Reference
*Caspase-3*	F: 5^\−^GAGCTTGGAACGGTACGCTA-3^\^ R: 5^\−^CCGTACCAGAGCGAGATGAC-3^\^	[26]
*Bax*	F: 5^\−^CGGCGAATTGGAGATGAACTGG-3^\^ R:5^\^CTAGCAAAGTAGAAGAGGGCAACC-3^\^	[27]
*GAPDH*	F: 5^\^AGCCTCGTCCCGTAGACAA-3^\^ R: 5^\^AATCTCCACTTTGCCACTGC-3^\^	[28]

The expression levels of *caspase-3* and *bax* genes in hepatic tissues were analyzed by qRT-PCR version 1.7 sequence detection software from PE Biosystems (Foster City, CA). The relative expression rate was estimated by calculating the difference in expression between the test gene and the reference gene (ΔCt) of the groups compared to that of the control group (ΔΔCt) and then calculating the fold change (2^−ΔΔCt^). All values were normalized to the reference gene (*GAPDH*).

### Histopathological Examination

Samples were taken from the liver of mice in different groups and fixed in 10% formalin for 24 h. Specimens were washed in tap water, dehydrated in ascending series of ethyl alcohol, cleared in xylene, embedded in paraffin wax, sectioned at 4 microns thickness by sledge microtome, and stained by hematoxylin and eosin stain for examination by light electric microscope [29].

### Statistical Analysis

Data were analyzed using the Statistical Package for the Social Sciences (SPSS 16) for Windows. The data distribution was tested by the Kolmogorov–Smirnov test. Data were analyzed using one-way analysis of variance (ANOVA) followed by Tukey’s test for multiple comparisons. The data were expressed as mean ± standard error of the mean (SEM). *P* < 0.05 was considered statistically significant.

## Results

### Hepatic Copper Accumulation

**Fig. 1:**
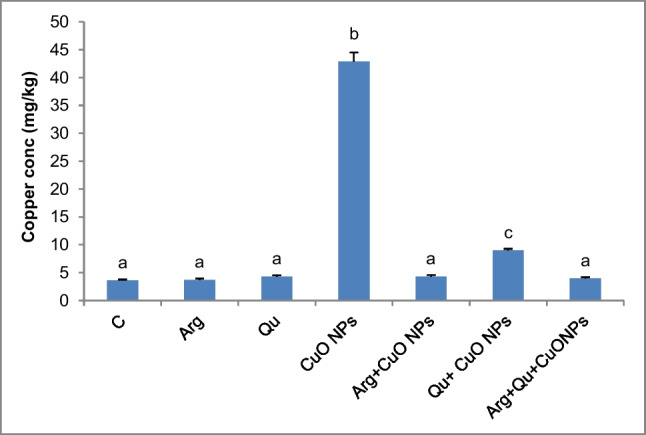
Copper levels in hepatic tissues of different studied groups. Data are expressed as mean $\pm$ SEM (*n* = 8). Different symbols are significant at *P* < 0.05 (C: control, Arg: l-arginine, Qu: quercetin, CuO NPs: copper oxide nanoparticles)

Administration of CuO NPs caused a significant elevation of the copper level in the hepatic tissue as compared to the control mice. This indicates the accumulation of CuO NPs in the hepatic cells. Treating mice with CuO NPs along with either l-Arg or Qu significantly prevented this accumulation of copper in the liver tissue compared to the CuO NP-intoxicated group (Fig. 1). Animals treated with arginine or the combined treatment with l-Arg and Qu demonstrated normal hepatic levels of copper comparable to that of the control group.

### Oxidative Stress and Redox Response

**Fig. 2:**
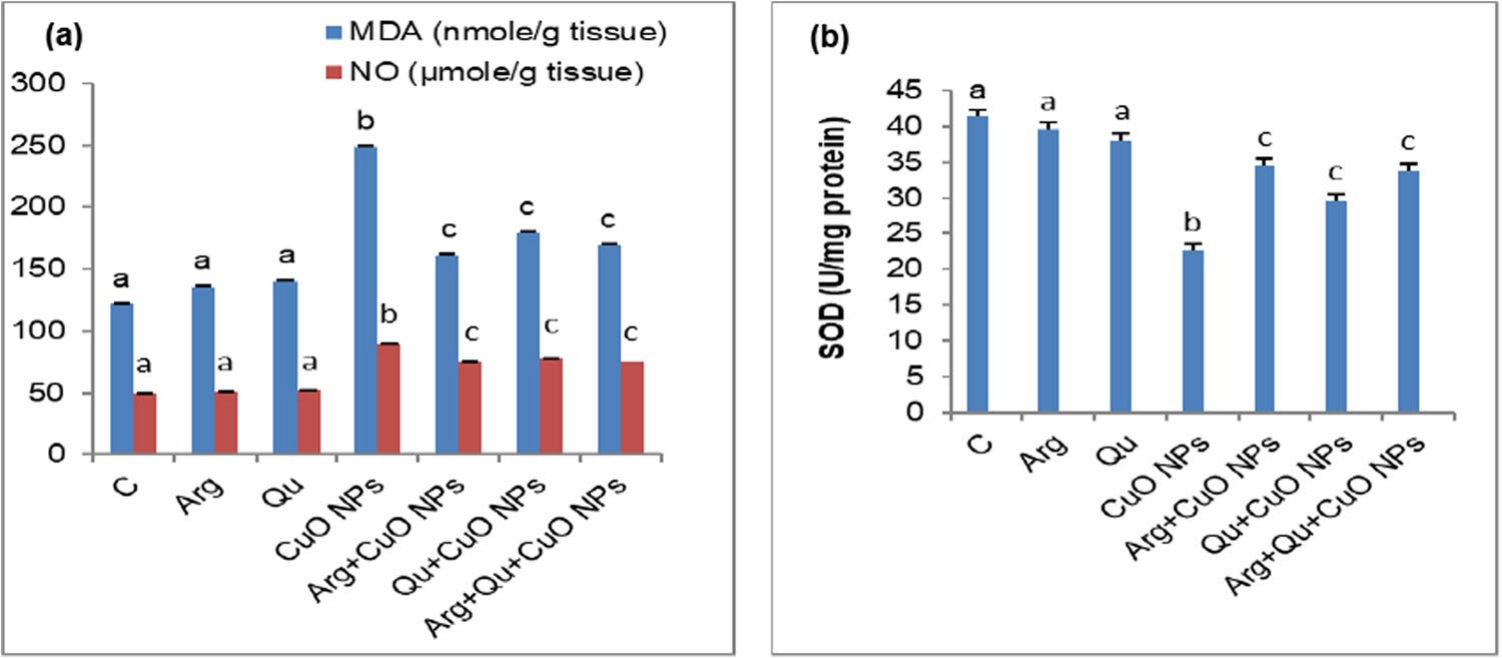
Effect of CuO NPs, Qu, and/or l-Arg on hepatic **a** MDA and NO levels and **b** SOD activity. Data are expressed as mean $\pm$ SEM (*n* = 8). Different symbols are significant at *P* < 0.05 (C: control, Arg: l-arginine, Qu: quercetin, CuO NPs: copper oxide nanoparticles)

Oral administration of CuO NPs to mice for 8 weeks induced hepatic oxidative stress and lipid peroxidation indicated by a significant (*P* < 0.05) increase in hepatic NO and MDA levels (Fig. 2a) which was accompanied by a significant (*P* < 0.05) decrease in SOD activity (Fig. 2b). Concomitant administration of CuO NPS with l-Arg and/or Qu significantly improved the redox state of liver tissue and prevented the elevation of MDA and NO levels and the reduction of SOD activity.

### Physiology and Functional Status of the Liver

**Fig. 3:**
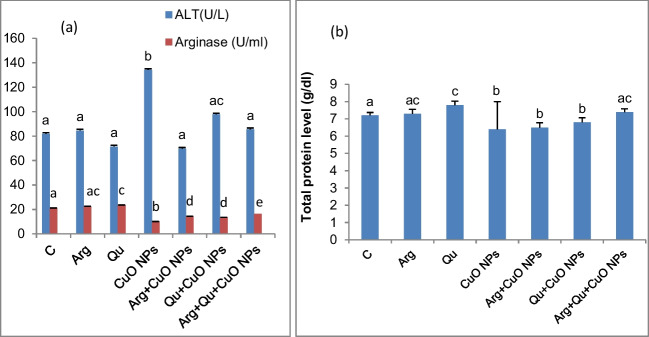
Effect of oral CuO NPs, Qu, and/or l-Arg on **a** serum ALT and hepatic arginase activities, **b** serum total protein. Data are expressed as mean $\pm$ SEM (*n* = 8) Different symbols are significant at *P* < 0.05 (C: control, Arg: l-arginine, Qu: quercetin, CuO NPs: copper oxide nanoparticles)

Oral administration of CuO NPs to mice induced hepatic injury indicated by a significant (*P* < 0.05) elevation of serum ALT accompanied by a significant (*P* < 0.05) reduction in the hepatic arginase activity (Fig. 3a) and serum total protein (Fig. 3b), compared to control mice. Co-administration of either l-Arg or Qu protected the mice from the deleterious effects of CuO NPs and significantly increased arginase activity (*P* < 0.05) and lowered serum ALT activity compared with CuO NP-intoxicated group (*P* < 0.05). Combined treatment with arginine and Qu normalized the changes in the arginase activity and was superior to each treatment alone. Although arginine or Qu prevented the reduction in the total protein level, however, this did not achieve statistical significance and only when the animals were administered with the combined Arg + Qu, the total protein level was normalized (Fig. 3b).

### Impact of CuO NPs, Qu, and/or l-Arg on Serum TNF-α

**Fig. 4:**
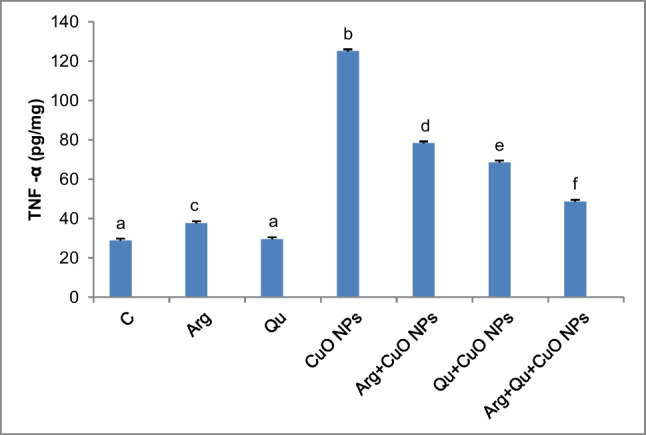
Changes of serum TNF-α levels in response to CuO NPs, Qu, and/or l-Arg. Data are expressed as mean $\pm$ SEM (*n* = 8). Different symbols are significant at *P* < 0.05 (C: control, Arg: l-arginine, Qu: quercetin, CuO NPs: copper oxide nanoparticles)

Sub-chronic exposure of experimental mice to CuO NPs resulted in liver tissue inflammation as seen by a significant (*P* < 0.05) rise in serum TNF-α level (Fig. 4). In contrast, mice treated with CuO NPs and received quercetin and/or l-arginine presented a considerable reduction in their serum TNF-α levels (*P* < 0.05) compared with the CuO NPs group. It is worth noting that the combination treatment resulted in a better effect in reducing serum TNF-α level (*P* < 0.05).

### Effect of CuO NPs, Qu, and/or L-Arg on the Expression Level of Caspase-3 & bax Genes in Liver Tissue

**Fig. 5:**
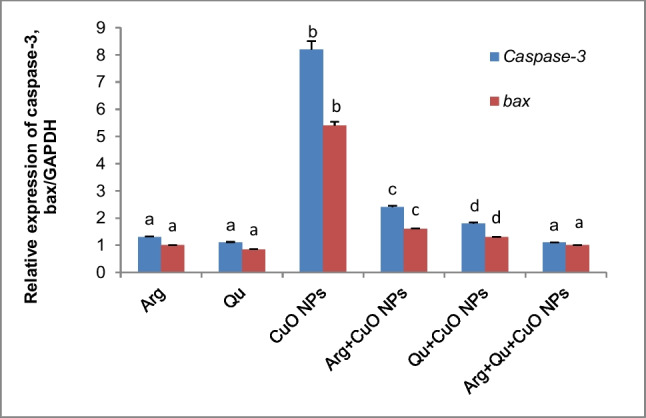
Effect of CuO NPs, Qu, and/or l-Arg on *caspase-3* and *bax* gene transcripts in liver tissue of all studied groups. Data are expressed as mean $\pm$ SEM (*n* = 6). Different symbols are significant at *P* < 0.05 (Arg: l-arginine, Qu: quercetin, CuO NPs: copper oxide nanoparticles)

Administration of CuO NPs to mice elevated the expression of apoptotic genes and caused a significant increase in the relative expression of hepatic *caspase-3 and bax* genes by ~ 8- and fivefold, respectively (Fig. 5). Whereas supplementing animals with CuO NPs with quercetin or l-arginine resulted in a marked reduction in the relative expression of the studied genes (*P* < 0.05). The combination treatment of CuO NP-administered mice with quercetin and l-arginine produced better results than individual supplements and normalized the gene expression level to the control level.

### Effect of CuO NPs, Qu, and/or l-Arg on Caspase-3 Activity in Liver Tissue

**Fig. 6:**
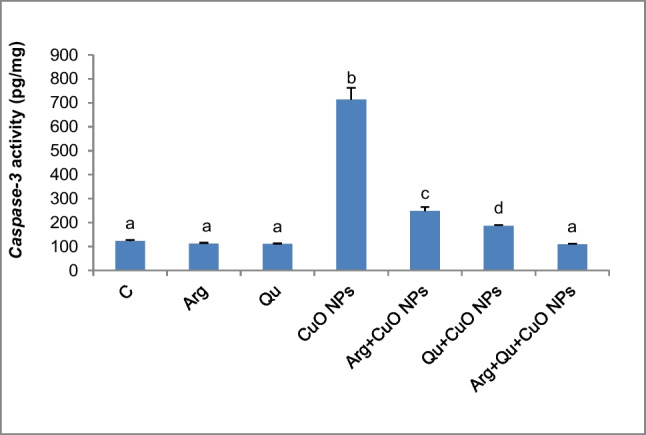
Caspase-3 activity in hepatic tissue of all experimental groups. Data are expressed as mean $\pm$ SEM (*n* = 8). Different symbols are significant at *P* < 0.05 (C: control, Arg: l-arginine, Qu: quercetin, CuO NPs: copper oxide nanoparticles)

Figure 6 demonstrates that animals treated orally with CuO NPs expressed higher activity of hepatic caspase-3 compared to the control group (*P* < 0.05), indicating hepatic apoptosis, and confirming the results of the gene expression. Co-administrating CuO NPs-treated mice with either l-Arg or Qu significantly prevented the elevation of caspase-3 activity by 65% and ~ 74%, respectively, compared to the CuO NPs group. Furthermore, the combination treatment with l-Arg and Qu to CuO NP group normalized the hepatic caspase-3 activity.

### Effect of Qu and/or L-Arg on CuO NP-Induced DNA Damage

**Fig. 7:**
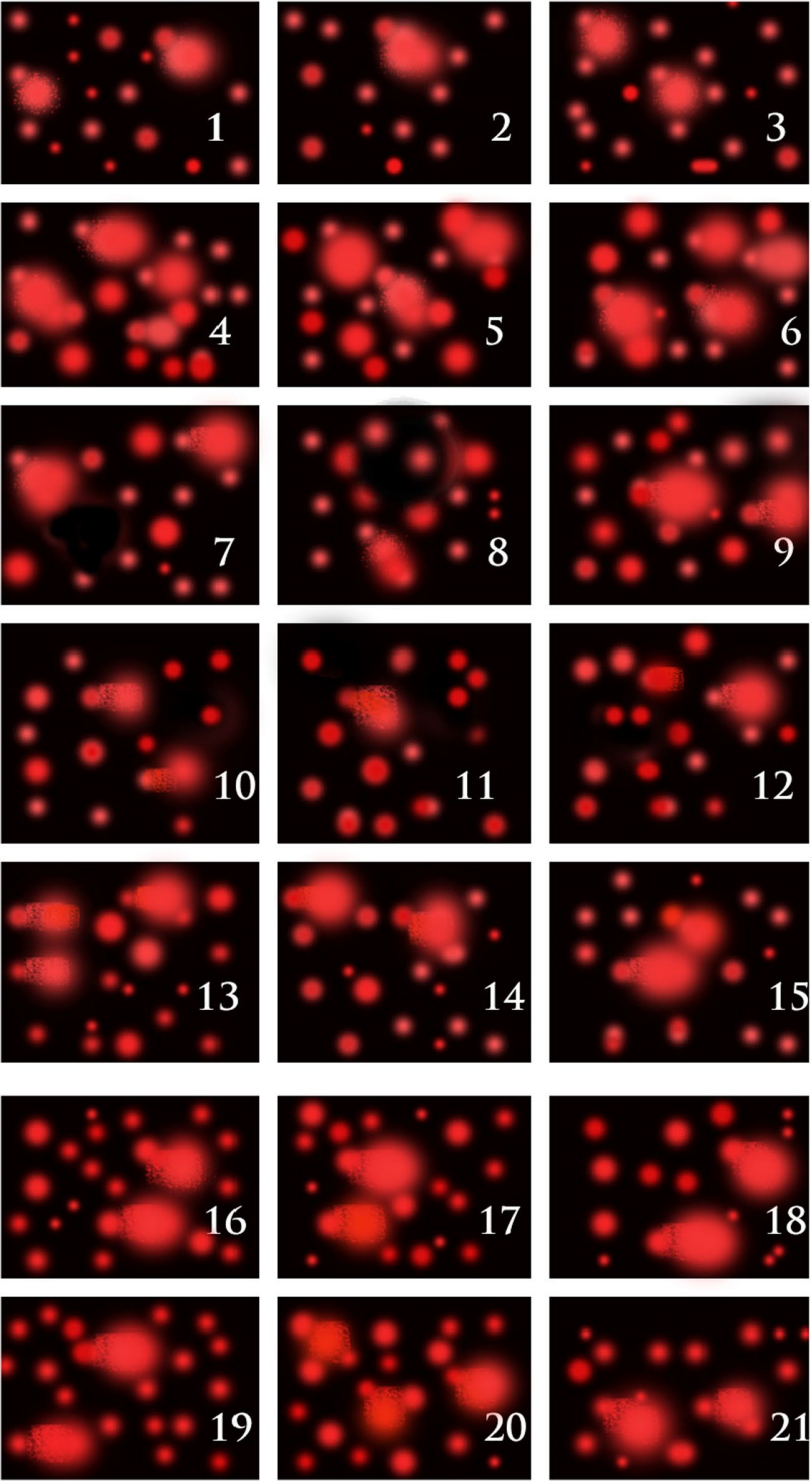
DNA fragmentation in liver tissues assessed by Comet assay. Three samples are presented for each group. Control group (1–3), CuO NP group (4–6), l-arginine group (7–9), quercetin group (10–12), and CuO NPs + l-Arg group (13–15). CuO NPs + Qu group (16–18), CuO NPs + l-Arg. + Qu group (19–21)

Administration of CuO NPs to mice resulted in a significant increase in DNA fragmentation assessed by Comet assay in liver tissue (tail DNA) by ~ 106% (Fig. 7), compared with the untreated group. Co-administrating CuO NP-treated mice with either Qu or l-Arg significantly reduced the tailed DNA by 15% and 9% respectively, while combination treatment of CuO NP-administered mice with Qu and l-Arg decreased tailed DNA by 21% compared with the CuO NP group.

### Histopathological Findings

**Fig. 8:**
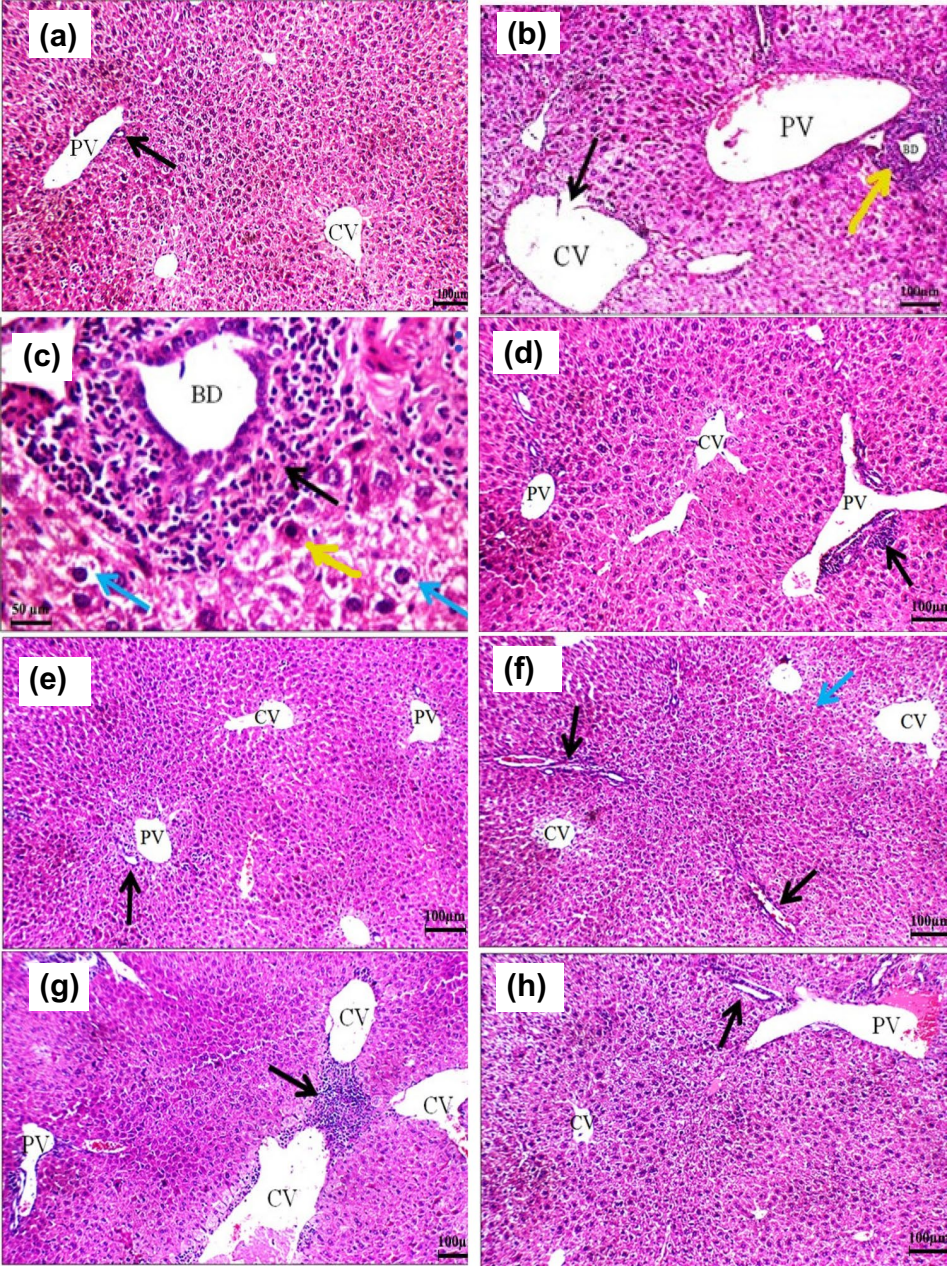
**a** Photomicrograph of a section in the liver of control mice showing average central veins (CV) with average portal tract, average portal vein (PV) and average bile duct (black arrow) (H&E × 200). **b** CuO NP-treated liver section showing a markedly dilated central vein (CV) with detached lining (black arrow) and expanded portal tract with markedly dilated portal vein (PV), average bile duct (BD), and marked portal inflammatory infiltrate (yellow arrow) (H&E × 200). **c** Liver from Cu ONPs group showing expanded portal tract with average bile duct (BD), marked portal inflammatory infiltrate (black arrow), and hepatocytes showing apoptosis (yellow arrow) and marked hydropic change I peri-portal area (blue arrows) (H&E × 400). **d** Liver of l-arginine group, showing average central vein (CV), and portal tract with dilated portal vein (PV), and mild portal inflammatory infiltrate (black arrow) (H&E × 200). **e** Liver section from quercetin group showing average central vein (CV), and average portal tracts with average portal vein (PV) and average bile duct (black arrow) (H&E × 200). **f** Photomicrograph of a section in the liver of CuO NPs + l-arginine group showing average central vein (CV), average portal tract (black arrows), and average hepatocytes (blue arrow) (H&E × 200). **g** Photomicrograph of a section in the liver of CuO NPs + quercetin group, showing dilated central vein (CV) with marked peri-venular inflammatory infiltrate (black arrow), and portal tract showing average portal vein (PV) (H&E × 200). **h** Photomicrograph of a section in the liver of CuO NPs + l-arginine + quercetin group presenting average central vein (CV), portal tract showing dilated congested portal vein (PV), and average bile duct (black arrow) (H&E × 200)

Histopathological abnormalities observed in the liver architecture of CuO NP-intoxicated mice (Fig. 8b and c) confirmed the liver injury and biochemical alterations reported. Photomicrographs of liver tissue from this group showed markedly dilated central veins with detached lining, expanded portal tracts with markedly dilated congested portal veins, and a marked portal inflammatory infiltrate, compared with the normal hepatic architecture of untreated mice (Fig. 8a). In addition, hepatocytes apoptotic and marked hydropic changes as well as areas of intra-lobular inflammatory infiltrate were detected (Fig. 8b and c). Liver sections from l-Arg-administered mice showed average central veins with slightly dilated portal veins and mild portal inflammatory infiltrate (Fig. 8d). Photomicrograph of liver sections from Qu-administered mice demonstrated average central veins with average portal veins and average bile ducts (Fig. 8e). Moreover, liver sections of l-Arg and CuO NP-administered mice showed average central veins with surrounding hepatocytes, mild hydropic changes, average portal tracts with average portal veins and average bile ducts, as well as average hepatocytes in peri-portal area (Fig. 8f), while liver sections from Qu and CuO NP-administered mice revealed dilated central veins with marked peri-venular inflammatory infiltrate, average portal tracts with average portal veins and mild hydropic changes in hepatocytes (Fig. 8g). Obviously, histopathological examination of liver sections of mice administrated with l-Arg, Qu, and CuO NPs showed average central veins, with dilated congested portal veins, average bile ducts, and mild hydropic changes in hepatocytes (Fig. 8h).

## Discussion

The widespread use and applications of CuO NPs in the daily life makes human exposure to these particles inevitable. When CuO NPs come into contact with living organisms, their nano-size, large surface area, and increased physicochemical reactivities can cause interactions with biomolecules causing serious cellular and tissue damage [30, 31]. The primary mechanism of CuO NP toxicity is attributed to the overproduction of reactive oxygen species (ROS), progression of oxidative stress, inflammation, biomolecule destruction, and genotoxicity. These effects occur when CuO NPs contact with the cell membrane and pass via tiny endocytic vesicle [32–34]. Excessive production of ROS is critical in the induction and progression of several diseases including liver disease [35]. In the current study, we investigated the role of quercetin and/or l-arginine in protecting or ameliorating the hepatotoxicity induced by copper oxide nanoparticles along 2-month duration period.

Regardless of their size, shape, dosage, or kind of substance, metal NPs typically accumulate in the liver. As indicated by the current findings, oral delivery of CuO NPs to mice (group 4) resulted in considerable copper buildup in the liver tissue causing hepatic injury. Our results are in agreement with previous studies [35, 36]. The higher bioaccumulation of copper in the liver is attributed to the liver fenestration and discontinuous endothelia which allow NPs of up to 100 nm to pass through blood into the parenchyma of the liver [37]. Treating the intoxicated animals with Qu and/or l-Arg led to a considerable decrease in the hepatic copper level. Qu and arginine could enhance copper elimination or bind copper preventing its accumulation in the liver. Quercetin has metallothionein induction and copper chelating properties [38]. Metallothionein is an endogenous metal chelator in enterocytes that has a higher affinity for copper than for zinc, causing it to bind luminal copper and preventing it from entering the circulation [39]. Arginine is a chiral α-amino acid with a tetrahedral stereo-center and a guanidine group forming a side chain that takes part in H-bonding and salt bridges [40]; thus, it can bind to Cu^+2^ ions forming Arg-Cu complex. Gao et al. [41] detected no increase in Cu^+2^ intracellular accumulations after Cu-Arg transport in Caco-2 cells. The authors related this observation to the high affinity of Cu^+2^ to amino acid residues in proteins.

Transition metals, including copper, are involved in ROS generation via the Fenton-type reaction mechanism, in which the metal ion reacts with H_2_O_2_ to produce hydroxyl radicals, which are extremely reactive and toxic to biological molecules, in addition to the oxidized metal ion itself [42]. CuO NPs consume hydrogen ions faster in the stomach and are converted into cupric ions with higher toxicity [43]. Once inside the mitochondria, CuO NPs increase ROS production by interfering with the electron transport chain, activating the NADPH-like enzyme system, and causing membrane depolarization due to damage to membrane phospholipids [44].

Nitric oxide (NO) and peroxynitrite are two examples of reactive oxygen and nitrogen species (ROS/RNS) that are hypothesized to be important pathophysiologic mediators of CuO NP-induced hepatotoxicity [45]. In the current study, the significant increase in hepatic NO level in the CuO NP-intoxicated group versus control animals is a sign of oxidative stress promotion in the liver tissue. This could be explained by the fact that CuO NPs increase inducible nitric oxide synthase 2 (iNOS2) activity in the liver, which in turn produces massive amounts of NO radical that interacts with oxygen radicals to generate peroxynitrite, a powerful oxidative and nitrosative agent that causes damage to hepatocytes [46]. Cells usually respond to oxidative burden by fortifying their antioxidant defense mechanism. However, the imbalance between oxidative burden and defense mechanism induces protein oxidation, lipid peroxidation, DNA damage, and apoptosis [35]. In the present study, the CuO NP-treated group presented a disturbance in the cell redox tone manifested by an increase in the MDA as well as a decrease in the antioxidant enzyme SOD in the liver tissue. It is widely accepted that SOD is a sensitive marker of liver damage as it scavenges superoxide anions to form H_2_O_2_ [47]. As interpreted by Gupta et al. [48], CuO NP-induced reduction in SOD protein was related to the ability of Cu ions to interact with the cysteine residues of SOD causing misfolding of the α-helix and β-sheet. Our results revealed that supplementing CuO NP-intoxicated mice with Qu and/or l-Arg effectively prevented the increase in the hepatic MDA and NO levels and increased the SOD activity. Qu has a potent inhibitory activity against the production of NO [49, 50]. Coinciding with our findings, Abdelhalim et al. [51] observed reduced levels of hepatic MDA in gold NP-treated rats administered with either Qu or l-Arg. Also, Liang et al. [52] demonstrated a dose-dependent depression of ROS and MDA levels with a significant elevation in the expression and activity of antioxidant enzymes in rats after oral administration with l-Arg. Supplementing CuO NP-treated mice with Qu effectively protected the hepatocytes by suppressing the ROS generation, scavenging lipid peroxidation products, and upregulating the activity of antioxidant enzymes such as SOD [53]. The free radicals scavenging activity of l-Arg was attributed to the electron transport mechanism, where l-Arg exerts its antioxidant activity by donating electrons to free radicals, thus terminating their chain reactions [52].

The liver has several crucial roles, including metal storage, metabolism, and detoxification [54]. Results of the current investigation show that mice given oral sub-chronic dose of CuO NPs demonstrated a large rise in ALT activity, which is a marker of liver injury. When the liver is injured, the hepatocyte transport function is impaired, which causes plasma membrane leakage and increases enzyme levels in the blood [50]. The liver produces total protein, and when the liver is damaged, production of these proteins is either diminished or entirely stopped. Total protein concentrations may provide information about the condition of the liver and the type of injury [55]. The liver also contains large amounts of arginase, an enzyme involved in the urea cycle that hydrolyzes l-arginine to l-ornithine and urea [56]. CuO NP-induced hepatocyte damage was also indicated by low levels of total protein and arginase in the intoxicated group in the current study implying the presence of hepatic insufficiency. In accordance, Lei et al. [3] found a dose-dependent increase in AST, ALP, ALT, and total bilirubin, as well as creatinine, lactate dehydrogenase, and BUN, whereas total protein and triglycerides levels declined significantly. On the contrary, non-significant alterations in serum biochemical liver and kidney parameters with mild transient minor liver damage were observed by Maciel-Magalhães et al. [57] on the day14, after exposing rats to a single acute oral dose of CuO NPs (2000 mg/kg). As presented by the current data, treating CuO NP-intoxicated mice with Qu, l-Arg, or their combination caused a significant reduction in ALT activity, as well as a significant elevation in total protein and arginase levels as compared to CuO NP-intoxicated group. The administration of these antioxidants prevented the deleterious effects of the copper nanoparticles and efficiently protected the hepatocytes. In accordance with our findings, Qu induced a significant reduction in the release of liver enzymes in cirrhotic rats induced by carbon tetrachloride [49]. This healing potency of quercetin may be attributed to its powerful antioxidant effect. Qu or l-Arg reduced levels of ALT and total protein in gold NP-treated rats [51]. This indicates that l-Arg and Qu prevented the lipid peroxidation of the cell membrane which resulted in the leakage of ALT. This seems to be the possible mechanism since both L-Arg and Qu reduced the MDA and NO, and elevated SOD activity in the current study. Additionally, l-Arg was found to enhance the antioxidant response and induce the synthesis of hepatic reduced glutathione (GSH) by stimulating the overexpression of hepatic arginase, thus providing l-glutamate, and activating nuclear factor erythroid 2-related factor 2 (Nrf2; a key transcription factor for antioxidant mechanism against oxidative stress pathway [52, 58]

Our results revealed that oral administration of CuO NPs to mice caused a significant elevation in serum TNF-α. In accordance, Sakr et al. [59] reported a marked elevation in splenic TNF-α in rats intoxicated with CuO NPs. The observed elevation in serum TNF-α levels may be due to the increase in ROS production and activation of nuclear factor-κB (NF-κB), a family of inducible transcription factors that mediates the induction of pro-inflammatory cytokines, such as TNF-α, IL-1, and IL-6 [60]. On the other hand, a significant reduction in TNF-α was detected in sera of mice groups supplemented with Qu, l-Arg, or their combination. Qu has a potent inhibitory activity against NO and TNF-α production in lipopolysaccharide-stimulated Kupffer cells [61]. Furthermore, Qu reduced inflammatory pain in Swiss mice by inhibiting oxidative stress and cytokine production, implying that Qu has anti-inflammatory properties in vivo [62]. l-Arg supplementation protects against oxidative damage and inflammatory responses caused by various pathological conditions [63]. Dietary l-Arg maintained normal liver morphology and function by suppressing the acute phase response of mast cells through the NO pathway and thus the production of TNF-α [64]. l-Arg inhibited the NF-kB DNA binding and stabilized the I-kB complex, which may account for the depletion of pro-inflammatory cytokines [65]. Much evidence suggests that the nutrient mixture of l-Arg and Qu can reduce the inflammatory response in experimentally induced inflammation by downregulating the expression of pro-inflammatory cytokines [14, 66]. The ability of these antioxidant agents to inhibit the expression of inflammatory mediators may explain their protective actions against CuO NP hepatotoxicity in mice.

There is a well-established link between NP-induced toxicity, DNA damage, lipid peroxidation, and oxidative stress, where the generated ROS react with DNA molecules causing damage to purine and pyrimidine bases as well as the DNA backbone [67]. As indicated in the present study, sub-chronic exposure of mice to CuO NPs resulted in massive genotoxicity and DNA damage. In agreement, Aziz and Abdullah [68] discovered genetic damage in *Labeo rohita* erythrocytes after 15, 30, and 45 days of treatment with 70.79 mg/L and 117.99 mg/L of 32.84 nm and rod shape CuO NPs. The highest genetic damage index was exhibited by the cells at the maximum dosage and treatment duration. In addition to the role of the generated oxidative stress in DNA damage following CuO NP exposure, damage of DNA can occur through the direct contact of CuO NPs with DNA, the DNA repair system, or DNA-related proteins, resulting in DNA breaks, deletions, mis-segregation, or nondisjunction [69]. Treating CuO NPs-intoxicated animals with Qu and/or l-Arg significantly protected against this DNA damage in accordance with previous studies [49, 65, 70]. Qu is well known for its capacity to reduce free radicals by donating hydrogen ions to them which lowers the generation of hydroxyl radicals or by scavenging ROS and decreasing DNA strand breaks and oxidized bases [71]. The antioxidant action of Qu is related to its ability to scavenge oxy radicals at several places throughout the lipid bilayer due to its pentahydroxyflavone structure, which allows it to bind metal ions via the orthodihydroxyphenolic arrangement [50]. As aforementioned, l-Arg’s antioxidant impact is achieved by scavenging free radicals via electron transport pathways and boosting the antioxidant defense system [52, 58]. However, the precise protective mechanism of action of l-Arg is unknown yet.

The ROS and genotoxicity caused by CuO NPs would lead to apoptosis by activating the mitochondrial apoptotic pathway, which involves overexpression of *Bax*, translocation of *Bax* into the mitochondrial membrane, and enhancement of caspase-3 activity. Caspase activation is critical in the execution of apoptosis. Our findings revealed a significant upregulation in the relative expression of hepatic *caspase-3* and *bax* genes as well as a significant elevation in caspase-3 activity in CuO NP-administered mice. Other previous studies have found that CuO NPs directly induce apoptosis by altering the expression of apoptotic genes [35, 72]. CuO NPs induce mitochondrial damage through the direct interaction of undissolved NPs with the ROS-derived lipid peroxides causing disruption of membrane integrity and release of apoptotic enzymes [73].

Supplementing CuO NP-intoxicated mice with l-Arg or Qu individually or in combination demonstrated a significant downregulation of the expression of *caspase-3* and *bax* genes and a significant reduction of the caspase-3 activity. This supports the anti-apoptotic properties of l-Arg and Qu. Furthermore, our observations coincide with the findings of Abdelazeim et al. [50] who demonstrated that Qu downregulated the expression of *bax* and inhibited the activity of caspase-3 in the liver of rats intoxicated with CuO NPs. In addition, l-Arg exerted a protective effect against necrosis and apoptosis caused by experimental ischemic and reperfusion in rat liver through the upregulation of BcL2 and NO synthesis [74].

By changing the membrane fluidity through ROS, damaging DNA, and inducing apoptosis, CuO NPs would have a detrimental effect on the architecture of the liver. The histopathological investigation revealed that sub-chronic oral administration of CuO NPs resulted in massive deterioration in the liver cells of mice which confirms the biochemical findings. This observation is consistent with a previous study [35]. Applying Qu and/or l-Arg with CuO NPs reduced the toxic effect of CuO NPs on the liver architecture. The combination treatment showed the most pronounced ameliorative effect. Qu significantly reduced the histopathological changes in the liver of copper oxide nanoparticles-treated rats in a previous study [49]. Our observations are also in line with the previous study of Sharma et al. [13] who demonstrated the hepatoprotective effect of l-Arg on paracetamol-intoxicated rats.

## Conclusion

Sub-chronic oral administration of CuO NPs to mice resulted in the bioaccumulation of copper in the liver. Copper produces free radicals that trigger oxidative insult, depletion of antioxidants, inflammation, DNA damage, liver injury, and apoptosis with a clear impact on the architecture of the liver. The study proved the efficiency of quercetin and l-arginine in mitigating CuO NP-induced liver toxicity due to their antioxidant and anti-inflammatory properties, ability to inhibit DNA damage, and the potential to act as good metal chelators.

## Data Availability

All data generated or analyzed during this study are included within the published article.

## References

[1] Crisan MC, Teodora M, Lucian M (2022). Copper nanoparticles: synthesis and characterization, physiology, toxicity and antimicrobial applications. Appl Sci.

[2] Naradala J, Allam A, Tumu VR, Rajaboina RK (2022). Antibacterial activity of copper nanoparticles synthesized by Bambusa arundinacea leaves extract. Biointerface Res Appl Chem.

[3] Lei R, Wu C, Yang B, Ma H, Shi C, Wang Q, Wang Q, Yuan Y, Liao M (2008). Integrated metabolomic analysis of the nano-sized copper particle-induced hepatotoxicity and nephrotoxicity in rats: a rapid in vivo screening method for nanotoxicity. Toxicol Appl Pharmacol.

[4] Gakis GP, Aviziotis IG, Charitidis CA (2023). Metal and metal oxide nanoparticle toxicity: moving towards a more holistic structure–activity approach. Environ Sci Nano.

[5] Ruiz P, Katsumiti A, Nieto JA, Bori J, Jimeno-Romero A, Reip P, Arostegui I, Orbea A, Cajaraville MP (2015). Short-term effects on antioxidant enzymes and long-term genotoxic and carcinogenic potential of CuO nanoparticles compared to bulk CuO and ionic copper in mussels *Mytilus*
*galloprovincialis*. Mar Environ Res.

[6] Midander K, Cronholm P, Karlsson HL, Elihn K, Möller L, Leygraf C, Wallinder IO (2009). Surface characteristics, copper release, and toxicity of nano-and micrometer-sized copper and copper(II) oxide particles: a cross-disciplinary study. Small.

[7] Sajjad H, Sajjad A, Haya RT, Khan MM, Zia M (2023). Copper oxide nanoparticles: In vitro and in vivo toxicity, mechanisms of action and factors influencing their toxicology. Comp Biochem Physiol C Toxicol Pharmacol.

[8] Xu D, Hu MJ, Wang YQ, Cui YL (2019). Antioxidant activities of quercetin and its complexes for medicinal application. Molecules.

[9] Jan AT, Kamli MR, Murtaza I, Singh JB, Ali A, Haq QMR (2010). Dietary flavonoid quercetin and associated health benefits- an overview. Food Nutr Res.

[10] Jung WJ, Sung MK (2004). Effects of major dietary antioxidant on inflammatory markers of RAW 264.7 macrophages. BioFactors.

[11] Gupta V, Gupta A, Saggu S, Divekar HM, Grover SK, Kumar R (2005). Anti-stress and adaptogenic activity of L-arginine supplementation. Evid-Based Complement Alternat Med.

[12] Ranjbar K, Nazem F, Nazari A (2015). Effect of exercise training and L-arginine on oxidative stress and left ventricular function in the post-ischemic failing rat heart. Cardiovasc Toxicol.

[13] Sharma V, Singh L, Shrivastav A, Verma N (2019). Effect of L-arginine amino acid on liver regeneration after hepatocyte damage in rats: an experimental study. J Drug Deliv Ther.

[14] Abdel Baky NA, Faddah LM, Al-Rasheed NM, Al-Rasheed NM, Shebali W (2013). Role of quercetin and L-arginine in alleviating zinc oxide nanoparticle hepatotoxicity in rats. Chiang Mai J Sci.

[15] Sharaf Eldin O, Bakry S, Abo Shaeir WA, Mohammed MS, Abd-Alzaher OF (2015). Possible hepatoprotective effect of quercetin against 2-butoxyethanol induced hepatic damage in rats. Middle-East J Sci Res.

[16] Beaty RD, Kerber JD (1993). Concepts, instrumentation and techniques in atomic absorption spectrophotometry. Perkin-Elmer Corp.

[17] Schumann G, Bonora R, Ceriotti F, Férard G, Ferrero CA, Frank PF, Gella FJ, Hoelzel W, Jørgensen PJ, Kanno T, Kessner A, Klauke R, Kristiansen N, Lessinger JM, Linsinger TP, Misaki H, Panteghini M, Pauwels J, Schiele F, Schimmel HG (2002). IFCC primary reference procedures for the measurement of catalytic activity concentrations of enzymes at 37°C. Clin Chem Lab Med.

[18] Burtis CA, Ashwood ER, Tietz NW (1999). Tietz textbook of clinical chemistry. Clin Chem.

[19] Augusto L, Amin PH, Wek RC, Sullivan WJ (2019). Regulation of arginine transport by GCN2 eIF2 kinase is important for replication of the intracellular parasite Toxoplasma gondii. PLoS Pathog.

[20] Nishikimi M, Roa NA, Yogi K (1972). The occurrence of superoxide anion in the reaction of reduced phenazine methosulfate and molecular oxygen. Biochem Biochem Biophys Res Commun.

[21] Ohkawa H, Ohishi W, Yagi K (1979). Assay for lipid peroxides in animal tissues by thiobarbituric acid reaction. Anal Biochem.

[22] Miranda KM, Espey MG, Wink DA (2001). A rapid simple spectrophotometric method for simultaneous detection of nitrate and nitrite. Nitric Oxide.

[23] Lau SY, Guild SJ, Barrett CJ, Chen Q, McCowan L, Jordan V, Chamley LW (2013). Tumor necrosis factor-alpha, interleukin-6, and interleukin-10 levels are altered in preeclampsia: a systematic review and meta-analysis. Am J Reprod Immunol.

[24] Rosen C, Nicholson DW, Chong T, Rowan KR, Thornberry NA, Miller DK, Rosen A (1996). Apopain/CPP32 cleaves proteins that are essential for cellular repair: a fundamental principle of apoptotic death. J Exp Med.

[25] Singh NP, McCoy MT, Tice RR, Schneider EL (1988). A simple technique for quantitation of low levels of DNA damage in individual cells. Exp Cell Res.

[26] Moazzen H, Venger K, Kant S, Leube RE, Krusche CA (2021). Desmoglein 2 regulates cardiogenesis by restricting hematopoiesis in the developing murine heart. Sci Rep.

[27] Chen Y, Sun J, Ju Z, Wang ZQ, Li T (2021). Nbs1-mediated DNA damage repair pathway regulates haematopoietic stem cell development and embryonic haematopoiesis. Cell Prolif.

[28] Agarwal N, Taberner FJ, Rangel Rojas D, Moroni M, Omberbasic D, Njoo C, Andrieux A, Gupta P, Bali KK, Herpel E, Faghihi F, Fleming T, Dejean A, Lechner SG, Nawroth PP, Lewin GR, Kuner R (2020). SUMOylation of enzymes and ion channels in sensory neurons protects against metabolic dysfunction, neuropathy, and sensory loss in diabetes. Neuron.

[29] Banchroft JD, Stevens A (1996) Theory and practice of histological techniques, 4th edn. Churchil Livingstone, New York

[30] Pourahmad J, Salami M, Zarei MH (2023). Comparative toxic effect of bulk copper oxide (CuO) and CuO nanoparticles on human red blood cells. Biol Trace Elem Res.

[31] Sajjad A, Bhatti SH, Ali Z, Jaffari GH, Khan NA, Rizvi ZF, Zia M (2021). Photoinduced fabrication of zinc oxide nanoparticles: transformation of morphological and biological response on light irradiance. ACS Omega.

[32] Mihailovic V, Katanic Stankovic J.S, Selakovic D, Rosic G (2021) An overview of the beneficial role of antioxidants in the treatment of nanoparticle-induced toxicities. Oxid Med Cell Longev 1–21. 10.1155/2021/7244677 10.1155/2021/7244677 PMC8608524 34820054

[33] Moschini E, Colombo G, Chirico G, Capitani G, Dalle-Donne I, Mantecca P (2023). Biological mechanism of cell oxidative stress and death during short-term exposure to nano CuO. Sci Rep.

[34] Tousson E, El-Gharbawy DM (2023). Impact of Saussurea lappa root extract against copper oxide nanoparticles induced oxidative stress and toxicity in rat cardiac tissues. Environ Toxicol.

[35] Ibrahim MA, Khalaf AA, Galal MK, Ogaly HA, Hassan AH (2015). Ameliorative influence of green tea extract on copper nanoparticle-induced hepatotoxicity in rats. Nanoscale Res Lett.

[36] Privalova L, Katsnelson BA, Loginova NV, Gurvich VB, Shur VY, Valamina IE, Makeyev OH, Sutunkova MP, Minigalieva IA, Kireyeva EP, Rusakov VO, Tyurnina AE, Kozin RV, Meshtcheryakova EY, Korotkov AV, Shuman EA, Zvereva AE, Kostykova SV (2014). Subchronic toxicity of copper oxide nanoparticles and its attenuation with the help of a combination of bioprotectors. Int J Mol Sci.

[37] Sadauskas E, Wallin H, Stoltenberg M, Vogel U, Doering P, Larsen A, Danscher G (2007). Kupffer cells are central in the removal of nanoparticles from the organism. Part Fibre Toxicol.

[38] Kuo SM, Huang CT, Blum P, Chang C (2001). Quercetin cumulatively enhances copper induction of metallothionein in intestinal cells. Biol Trace Elem Res.

[39] Tapiero H, Townsend DM, Tew KD (2003). Trace elements in human physiology and pathology. Copper Biomed Pharmacother.

[40] Fitch CA, Platzer G, Okon M (2015). Arginine: its pKa value revisited. Protein Sci.

[41] Gao S, Yin T, Xu B, Ma Y, Hu M (2014). Amino acid facilitates absorption of copper in the Caco-2 cell culture model. Life Sci.

[42] Valavanidis A, Vlachogianni T, Fiotakis C (2009). 8-Hydrox2′ deoxyguanosine (8-OHdG): a critical biomarker of oxidative stress and carcinogenesis. J Environ Sci Health C Environ Carcinog Ecotoxicol Rev.

[43] Meng H, Chen Z, Xing GM, Yuan H, Chen CY, Zhao F, Zhang C, Wang Y, Zhao Y (2007). Ultra high reactivity and grave nanotoxicity of copper nanoparticles. Toxicol Lett.

[44] Lenaz G (2001). The mitochondrial production of reactive oxygen species: mechanisms and implications in human pathology. IUBMB Life.

[45] Stine JG, Chalasani N (2015). Chronic liver injury induced by drugs: a systematic review. Liver Int.

[46] Bartsch H, Nair J (2006). Chronic inflammation and oxidative stress in the genesis and perpetuation of cancer: role of lipid peroxidation, DNA damage, and repair. Langenbecks Arch Surg.

[47] El-Maraghi EF, Abdel-Fattah KI, Soliman SM, El-Sayed WM (2020). Taurine abates the liver damage induced by γ-irradiation in rats through anti-inflammatory and anti-apoptotic pathways. Int J Rad Biol.

[48] Gupta G, Cappellini F, Farcal L, Gornati R, Bernardini G, Fadeel B (2022). Copper oxide nanoparticles trigger macrophage cell death with misfolding of Cu/Zn superoxide dismutase 1 (SOD1). Part Fibre Toxicol.

[49] Arafa AF, Ghanem HZ, Soliman MS, EL-Meligy E,  (2017). Modulation effects of quercetin against copper oxide nanoparticles-induced liver toxicity in rats. Egypt Pharmaceut J.

[50] Abdelazeim SA, Shehata NI, Aly HF, Shams SG (2020). Amelioration of oxidative stress-mediated apoptosis in copper oxide nanoparticles-induced liver injury in rats by potent antioxidants. Sci Rep.

[51] Abdelhalim MA, Moussa SA, Qaid HA (2018). The protective role of quercetin and arginine on gold nanoparticles induced hepatotoxicity in rats. Int J Nanomedicine.

[52] Liang M, Wang Z, Li H, Cai L, Pan J, He H, Wu Q, Tang Y, Ma J, Yang L (2018). L-Arginine induces antioxidant response to prevent oxidative stress via stimulation of glutathione synthesis and activation of Nrf2 pathway. Food Chem Toxicol.

[53] Abdeltawab AR, Elsyyad HI, Abdelaziz KK, El-Beltagy AB (2021). Therapeutic role of quercetin against experimentally induced hepatocellular carcinoma in female albino rats and their offspring. JBAAR.

[54] Tao TY, Gitlin JD (2003). Hepatic copper metabolism: insights from genetic disease. Hepatology.

[55] Chukwudoruo C, Sunday C, Ibe OK, Chinenyenwa N, Igwe OK, Iheme IC, Ujowundu FN, Mba BA (2021). Serum total protein concentration and liver enzymes activities in albino rats model administered with ethanolic leaf extract of *Ficus*
*capensis*. A Jof B.

[56] Ikemoto M, Tsunekawa S, Awane M, Fukuda Y, Murayama H, Igarashi M, Nagata A, Kasai Y, Totani M (2001). A useful ELISA system for human liver-type arginase, and its utility in diagnosis of liver diseases. Clin Biochem.

[57] Maciel-Magalhães M, Medeiros RJ, Bravin JS, Patricio BFC, Rocha HVA, Paes-de-Almeida EC, Santos LMG, Jacob SC, Savignon TCM, Amendoeira FC (2020). Evaluation of acute toxicity and copper accumulation in organs of Wistar rats, 14 days after oral exposure to copper oxide (II) nano- and microparticles. J Nanopart Res.

[58] Nguyen T, Nioi P, Pickett CB (2009). The Nrf2-antioxidant response element signaling pathway and its activation by oxidative stress. J Biol Chem.

[59] Sakr S, Abdelwahab M, Atef M (2021). The oxidative stress mediated toxicity and immunotoxic effect of copper oxide nanoparticles on spleen of adult albino rats. ZJFMT.

[60] Liu T, Zhang L, Joo D, Sun SC (2017). NF-κB signaling in inflammation. Signal Transduct Target Ther.

[61] Kawada N, Seki S, Inoue M, Kuroki T (1998). Effect of antioxidants, resveratrol, quercetin, and N-acetylcysteine, on the functions of cultured rat hepatic stellate cells and Kupffer cells. Hepatology.

[62] Valerio DA, Georgetti SR, Magro DA, Casagrande R, Cunha TM, Vicentini FT, Viera SH, Fonseca MJ, Ferreira SH, Cunha FQ, Verri WA (2009). Quercetin reduces inflammatory pain: Inhibition of oxidative stress and cytokine production. J Nat Prod.

[63] Huang CC, Tsai SC, Lin WT (2008). Potential ergogenic effects of L-Arg against oxidative and inflammatory stress induced by acute exercise in aging rats. Exp Gerontol.

[64] Li Q, Liu Y, Che Z, Zhu H, Meng G, Hou Y, Ding B, Yin Y, Chen F (2012). Dietary L-arginine supplementation alleviates liver injury caused by Escherichia coli LPS in weaned pigs. Innate Immun.

[65] Calkins CM, Bensard DD, Heimbach JK, Meng X, Shames BD, Pulido EJ, McIntyre RC (2001). L-Arginine attenuates lipopolysaccharide- induced lung chemokine production. Am J Physiol Lung Cell Mol Physiol.

[66] Ivanov V, Ivanova JCS, Kalinovsky T, Roomi MW, Rath M, Niedzwiecki A (2008). Essential nutrients suppress inflammation by modulating key inflammatory gene expression. Int J Mol Med.

[67] Al-Hamadani MY, Alzahrani AM, Yousef M, Kamel MA, El-Sayed WM (2020). Gold nanoparticles perturb drug metabolizing enzymes and antioxidants in the livers of male rats: potential impact on drug interactions. Int J Nanomed.

[68] Aziz S, Abdullah S (2023). Evaluation of toxicity induced by engineered CuO nanoparticles in freshwater fish, Labeo rohita. Turk J Fish Aquat Sci.

[69] Dube E, Okuthe GE (2023) Engineered nanoparticles in aquatic systems: toxicity and mechanism of toxicity in fish. Emerg Contam 100212.10.1016/j.emcon.2023.100212

[70] Faddah LM, Abdel-Baky NA, Al-Rasheed NM, Al-Rasheed NM (2003). Biochemical responses of nanosize titanium dioxide in the heart of rats following administration of idepenone and quercetin. Afr J Pharm Pharmacol.

[71] Wilms LC, Hollman PC, Boots AW, Kleinjans JC (2005). Protection by quercetin and quercetin rich fruit against induction of oxidant DNA damage and formation of BPDE-DNA adducts in human lymphocytes. Mutat Res.

[72] Sarkar A, Das J, Manna P, Sil C (2011). Nano-copper induces oxidative stress and apoptosis in kidney via both extrinsic and intrinsic pathways. Toxicology.

[73] Semisch A, Ohle J, Witt B, Hartwig A (2014). Cytotoxicity and genotoxicity of nano - and microparticulate copper oxide: role of solubility and intracellular bioavailability. Part Fibre Toxicol.

[74] Chattopadhyay P, Shukla G, Wahi AK (2009). Protective effect of L-arginine against necrosis and apoptosis induced by experimental ischemic and reperfusion in rat liver. Saudi J Gastroenterol.

